# Polybacterial Periodontal Infection Alters oxidative and Inflammatory Biomarkers in Primary Human Aortic Endothelial Cell (pHAECs)

**DOI:** 10.26502/fjppr.0108

**Published:** 2025-04-19

**Authors:** Chethan Sampath, Michaela Campbell, Jonathan Baptiste, Jalah Carter, Mia-Sade Greene, Gunaraj Dhungana, Sasanka Chukkapalli, Pandu R Gangula

**Affiliations:** 1 Department of Oral Diagnostic Sciences & Research, Department of Periodontology, School of Dentistry, Meharry Medical College, Nashville, TN 37208, USA; 2 Department of Biomedical Engineering, Texas A & M University, College Station, TX 77843, USA

**Keywords:** polybacterial periodontal pathogens, nitric oxide, primary human aortic endothelial cells, nitric oxide synthase

## Abstract

**Background:**

Periodontitis (PD) is a highly prevalent inflammatory disease associated with complex microbial infection in the subgingival cavity. We have previously shown that polybacterial periodontal infection led to aortic atherosclerosis and lipid profile modulation; however, the underlying mechanism(s) has not been yet demonstrated.

**Methods:**

Primary human aortic endothelial cells (pHAECs) were infected periodically for 48 hours either with *P. gingivalis* (monobacterial infection) or polybacterial periodontal pathogens, *P. gingivalis* (*Pg*), *T. forsythia* (*Tf*), *T. denticola* (*Td*), and *F. nucleatum* (*Fn*), using HoxBan coculture technique. Cell viability was assessed using MTT assay. Nitric oxide synthesis was measured in the form of total nitrite released into the media after incubation period. Inflammatory and oxidative gene expression were evaluated in the cell lysate using quantitative real-time polymerase chain reaction (qRT-PCR) at each time point (12-48h). Statistical analysis was performed using two-way ANOVA.

**Results:**

pHAEC cell viability was significantly (p<0.05) reduced in both mono and poly-bacterial infection when compared with non-infected cells in a time dependent manner. Nitrite levels in the media were significantly impaired in both infection groups. mRNA expression for cytokines (TLR-4, IL-6, and TNF-α,) and inducible nitric oxide synthase (iNOS) were significantly (p<0.05) elevated in both experimental groups. In contrast, endothelial (e) NOS, tetrahydrobiopterin (BH_4_) synthesis, GCH −1, Nrf2, and Nqo 1 were significantly (p<0.05) reduced in both experimental groups. Finally, polybacterial infection group showed greater changes in cell viability, nitrite levels, cytokines, eNOS/BH_4_/Nrf2 expression in a time dependent manner.

**Conclusions:**

Our study highlights the adverse effects of *in vitro* PD infection in NO mediated vascular endothelial cell function.

## Introduction

Periodontal disease (PD),is characterized by gingival inflammation and the destruction of periodontal tissues [1]. PD is among the most common chronic infections affecting up to an estimated 20–50% of the global population [2]. PD is not solely caused by a single or a few select organisms but by polymicrobial dysbiosis [3]. The red complex group bacteria involved in periodontitis constitutes *Porphyromonas gingivalis* (*Pg*), *Tannerella forsythia* (*Tf*), and *Treponema denticola* (*Td*) and a bridge complex-forming bacterium, *Fusobacterium nucleatum (Fn)* [4]. Periodontal infection is not only contributes to poor oral health but also implicated in multiple systemic diseases, such as hypertension, atherosclerosis, stroke and diabetes [5]. A healthy vascular endothelium is essential for the maintenance of cardiovascular homeostasis and the prevention of atherosclerosis [6]. Endothelial dysfunction is an early pathogenic event for atherosclerosis, hypertension, and various other cardiovascular disorders [7]. Endothelial cells generate and secrete a variety of signaling molecules that influence cardiovascular physiology by the regulation of blood clotting, maintaining blood vessel size, cellular permeability, inflammation, and angiogenesis [7]. Among these substances, nitric oxide (NO) is a key molecule that significantly influences the physiology of the endothelium and endothelial dysfunction is often simply defined as diminished NO bioavailability [8]. In endothelial cells, NO is produced by endothelial nitric oxide synthase (eNOS), whereas neuronal NOS (nNOS) expressed primarily in neurons [9]. Both e- and n- NOS are constitute and calcium dependent. Conversely, iNOS, is expressed in various cell types during infection and in response to inflammatory stimuli, such as IL-1, TNF-*α*, IFN-*γ* and LPS, preferably in a synergic manner, yielding high amounts of NO for a longer period of time [10]. In addition, nuclear factor erythroid 2-related factor (Nrf2) is known to interact with NO and play a vital role in regulating antioxidant genes in various cell types. However, to date, no studies have been reported the impact of polybacterial periodontal pathogens in vascular endothelial function *in vitro* and how does it comparable with mono infection with *Pg*. Hence, in this study we examined the effects of mono (*Pg*) vs polybacterial infections in primary human aortic endothelial cell function as measured by changes in cell viability, BH_4_/NO/Nrf2 synthesis, inflammation and oxidative stress markers.

## Materials and Methods

### Pre-culture of bacteria

In this study, *P. gingivalis* strain (W83), *T. denticola* (*Td*, ATCC 35404), *T. forsythia* (*Tf*, ATCC 43037), and *F. nucleatum* (*Fn*, ATCC 49256) were cultured individually in the anaerobic chamber (ATCC, Rockville, MD, USA) at 37°C[11]. The bacterial concentrations were measured (OD_600_ of ≈ 0.8).

### Pre-culture of primary human aortic endothelial cells (pHAECs)

*pHAECs (*PCS-100-011, ATCC, Rockville, MD) were cultured in a vascular cell basal medium that was supplemented with an Endothelial Cell Growth Kit-VEGF according to manufacturer’s protocol (ATCC, Rockville, MD). The cells were maintained in a humidified incubator at 37°C with 5% CO_2_ and were seeded at approximately 50% confluency in a 12-well plate with coverslips. All experiments utilized confluent cells from the fourth to sixth passage. All reagents were obtained from ATCC or Fisher Scientific.

### Protocol for the Human oxygen-Bacteria anaerobic (HoxBan) coculture

The Human oxygen-Bacteria anaerobic (HoxBan) coculture method was conducted with minor modifications and as previously described [12]. Specifically, 1 milliliter of an overnight *Pg*, *Td*, *Tf*, and *Fn,* 10^9^ colony-forming units) pre-culture was used to inoculate 1,000 mL of freshly autoclaved and cooled (≈ 40°C) OTEB broth containing 1% agar. 40 milliliter aliquots of this inoculum were then transferred into sterile Falcon 50 mL conical centrifuge tubes and allowed to solidify for 30 minutes. The *Pg* alone and the poly-inoculated Falcon tube cultures were subsequently placed in a tissue culture cabinet with ambient air. pHAECs (≈ 5,000 cells) cultured on coverslips were positioned upside down on top of the solidified agar and covered with 10 mL of pre-warmed (37°C) DMEM medium (without antibiotics). For the control conditions (uninfected), *pHAECs* cultured on coverslips were placed upside down on the agar without mono or polybacteria. After setting up the cultures, the coculture tubes were incubated in a humidified incubator at 37°C and 5% CO_2_ for various time points ranging from 0 to 48 h. The multiplicity of infection (MOI) was calculated based on the number of cells per well at seeding. This experiment was repeated three times, with each condition performed in duplicate.

### Cell Viability Assay

MTT [3-(4,5-Dimethylthiazol-2-yl)-2,5-Diphenyltetrazolium Bromide] assay was performed to assess the toxicity of the pHAECs [13]. At the end of each time point 50 μl of 5 mg/mL MTT reagent was added to the cells. The cells were subsequently incubated for 4 h at 37 °C until formation of formazan crystals. The formazan crystals were quantitated by dissolving in 500 μl of DMSO (Sigma Aldrich, St. Louis, MO) and the absorbance was read at 570 nm using microplate reader (BioTek, Winooski, VA).

### Nitric Oxide Determination

In brief, at the end of the co-culture experiment for each time point as described in the above sections, the cell culture supernatants were collected for nitric oxide (NO) release using a NO assay kit (Biovision, Milpitas, CA). The assay was performed according to the manufacturer’s protocol.

### RNA isolation and quantitative real-time polymerase chain reaction (PCR) analysis

At the end of the co-culture experiment, pHAECs adherent to coverslips were removed from co-culture tubes, and total RNA of cells was extracted with TRIzol. The quality of RNA was determined by NanoDrop (Thermofisher Scientific, Waltham, MA). RNA was reverse-transcribed to cDNA using iScript cDNA Synthesis Kit (Bio-Rad, Hercules, CA). The primers designed to detect various biomarkers were depicted in Table 1. Quantitative real time-polymerase chain reaction assay was performed using SYBR Green (Bio-Rad, Hercules, CA). Relative expression levels of target genes were normalized to β-actin, and threshold cycle (Ct) numbers were calculated using 2^−∆∆CT^ method). All studies were performed in the Meharry Medical College Molecular Biology Core Laboratory.

### Statistical analysis

Statistical analysis was conducted using GraphPad Prism (GraphPad Software, version 9, Boston, MA). Student t-test or two-way ANOVA was employed to assess significant differences between groups. A p-value of less than 0.05 was considered statistically significant. ^*a*^ p< 0.05 compared with control (uninfected) cells. ^*b*^ p< 0.05 compared with monobacterial infection.

## Results

### Polybacterial periodontal pathogens inhibits pHAEC viability

**Figure 1:**
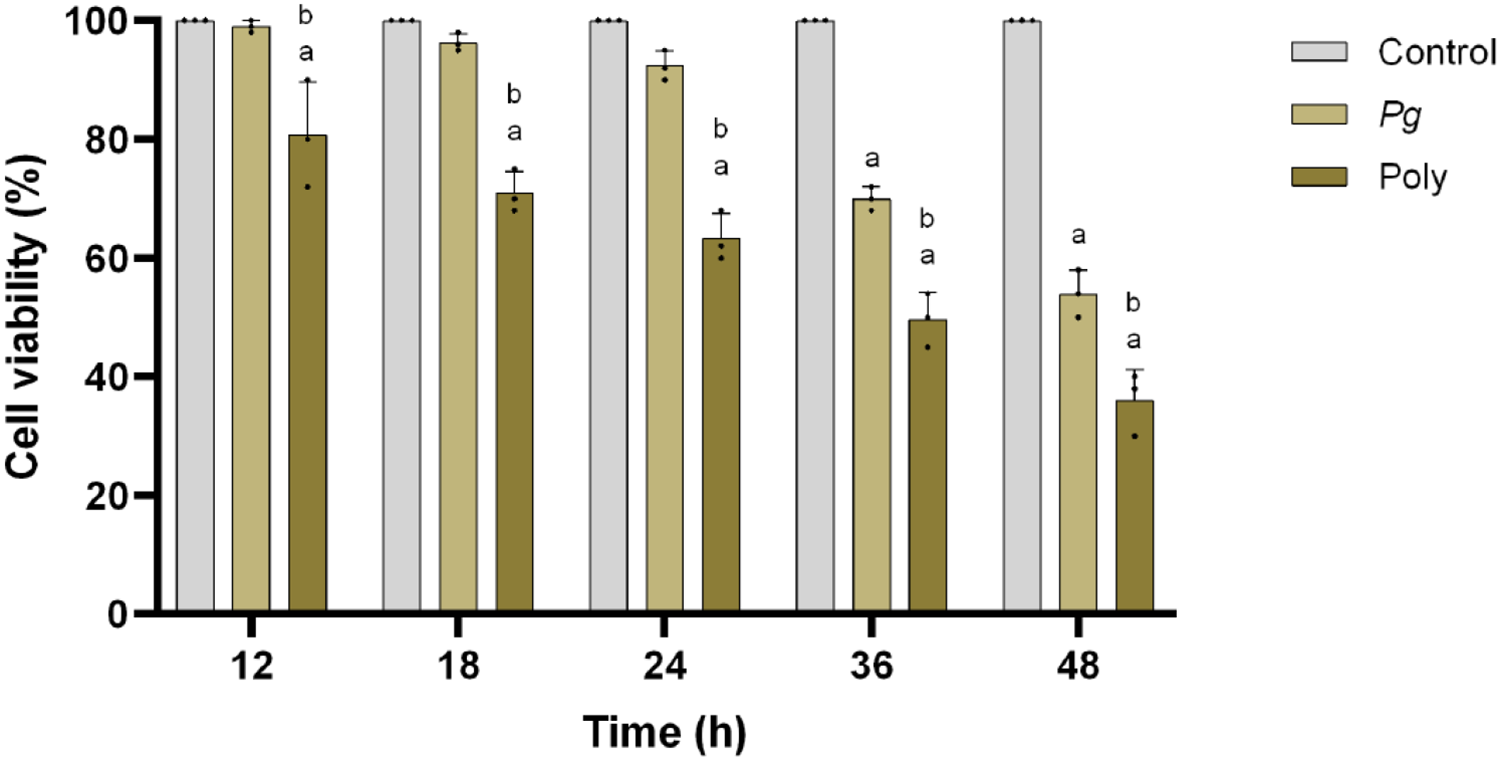
Mono and polybacterial cytotoxicity against pHAEC’s. The cell growth inhibitory rate was measured using MTT assay. The values are expressed as percentage of the control (uninfected with mono or polybacterial) at each time point, respectively (0 to 48 h). The results are from three independent experiments are represented in the form of means ± SD (n = 3, three independent experiments and, in each experiment, we had duplicates values for all the results). ^*a*^ p< 0.05 compared with control (uninfected) cells. ^*b*^ p< 0.05 compared with monobacterial infected cells. *Pg*—*P. gingivalis*; Poly – polybacterial.

To evaluate the potential inherent toxicity of the mono and polybacterial infection, the pHAECs were co-cultured for different time intervals (12–48 h). Cell viability was determined using an MTT assay. As shown in Fig 1., of all the time points tested, polybacterial periodontal infection showed greater reduction of cell viability compared to mono infection with *Pg* in a time dependent manner. Monobacterial (*Pg*) infection did not affect on cell viability when compared to that of polybacterial infection at 12h. Polybacterial infection reduced cell viability to around 20% at 12 h and to almost 55 % at 48 h.

### Effect of mono and polybacterial infection on NO synthesis

**Figure 2:**
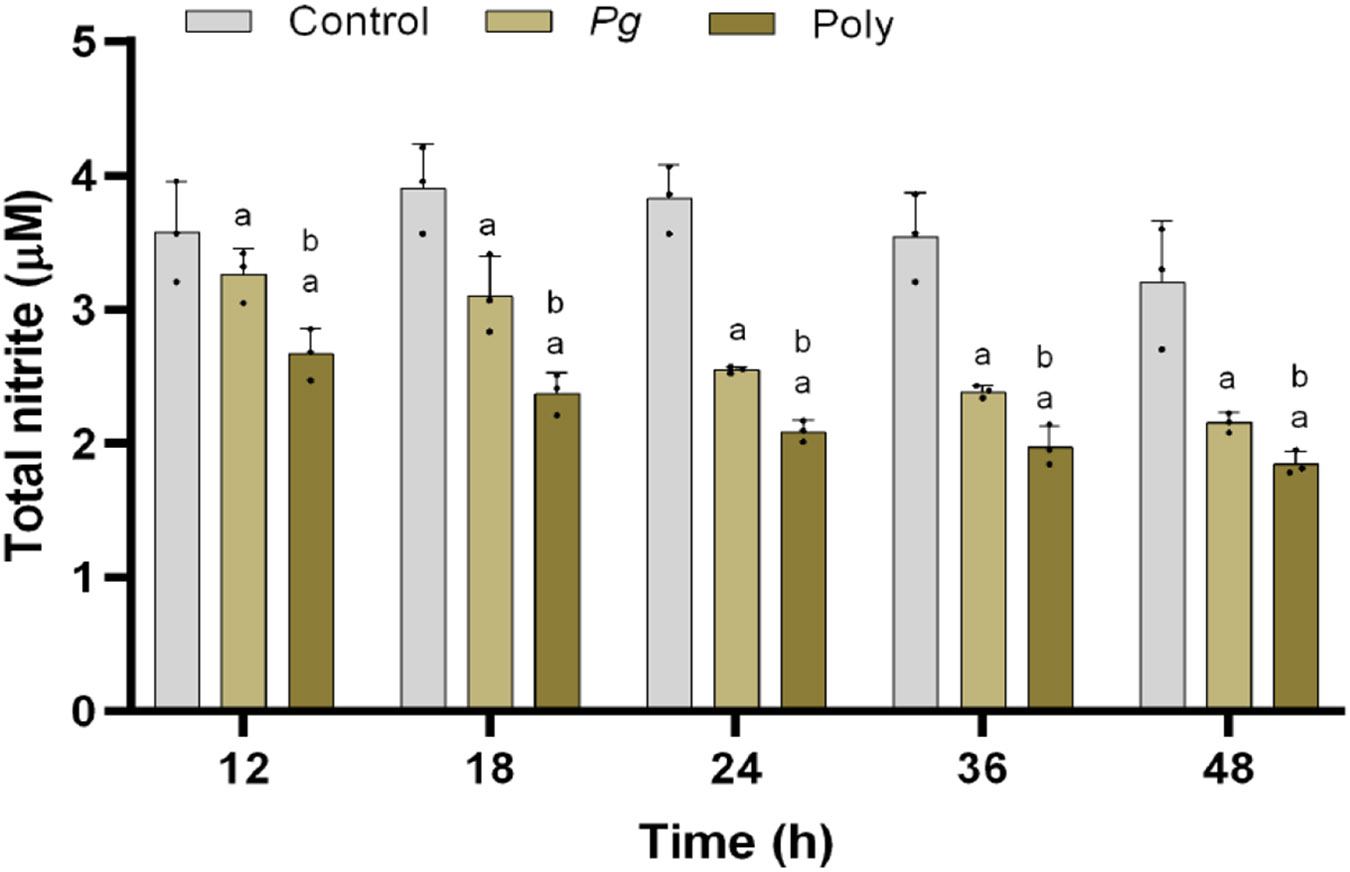
Mono and polybacterial induced nitric oxide changes in pHAEC’s. The nitric oxide concentration in the supernatant at each time point (0 to 48 h), respectively was determined by the Griess reaction. The results are from three independent experiments are represented in the form of means ± SD (n = 3, three independent experiments and, in each experiment, we had duplicates values for all the results). ^*a*^ p< 0.05 compared with control (uninfected) cells. ^*b*^ p< 0.05 compared with monobacterial infected cells. *Pg*—*P. gingivalis*; Poly – polybacterial.

Nitric oxide (NO) plays a vital role in endothelium mediated vascular function [14]. As shown in figure 2, pHAECs co-cultured with *Pg* or polybacterial periodontal pathogens demonstrated a significant reduction in nitrite production when compared with the control (uninfected) group in a time dependent manner. Cells exposed to polybacterial infection showed greater reduction compared to mono-infection and the control (Fig. 2). This suggests that PD infection impairs nitrite production potentially influencing cardiovascular health.

### Differential expression of iNOS and eNOS with periodontal pathogen infection

**Figure 3:**
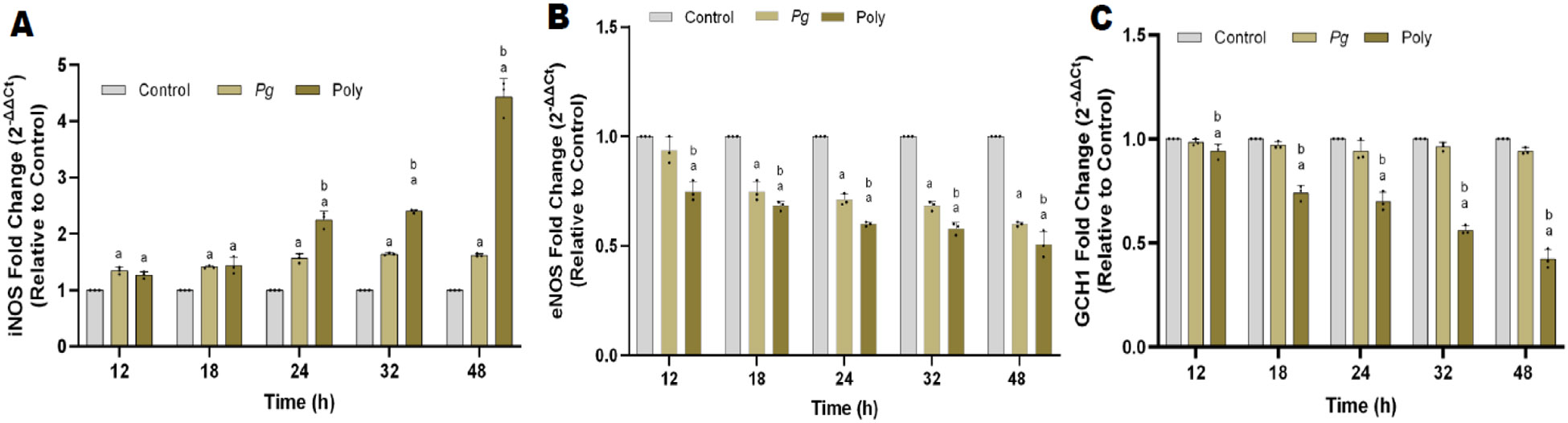
Time-dependent effect of mono and polybacterial infection on mRNA expressions. (**A**) iNOS, (**B**) eNOS and (**C**), GCH-1, mRNA expressions in pHAECs at each time point (0 to 48 h), respectively. Data were normalized with housekeeping gene (*β*-actin). All data are represented as 2^−ΔΔCt^ relative to vehicle. The results are from three independent experiments are represented in the form of means ± SD, three independent experiments and, in each experiment, we had duplicates values for all the results). ^*a*^ *p*< 0.05 compared with control (uninfected) cells. ^*b*^ *p*< 0.05 compared with monobacterial infected cells. *Pg*—*P. gingivalis*; Poly – polybacterial; iNOS - inducible nitric oxide synthase; eNOS - endothelial nitric oxide synthase; GCH-1 - GTP cyclohydrolase-1.

The enzymatic formation of NO is catalyzed by nitric oxide synthase (NOS) through a series of redox reactions. The mRNA expression of iNOS significantly increased with *Pg* and polybacterial infection when compared to the control (uninfected) group in all time points tested (Fig. 3A). In contrast, polybacterial infection exerted a four-fold higher iNOS expression than *Pg* alone at 48 h time (Fig. 3A). In contrast, *Pg* and polybacterial infection reduced eNOS levels in a time-dependent manner (Fig. 3B). Under physiological conditions, GTPCH (GCH-1) is the rate-controlling enzyme in the BH_4_ pathway. The mRNA expression showed a significant (p<0.5) decrease in GCH-1 in polybacterial but not in mono bacterial infected group (Fig. 3C).

### Polybacterial infection increased inflammatory cytokine production

**Figure 4:**
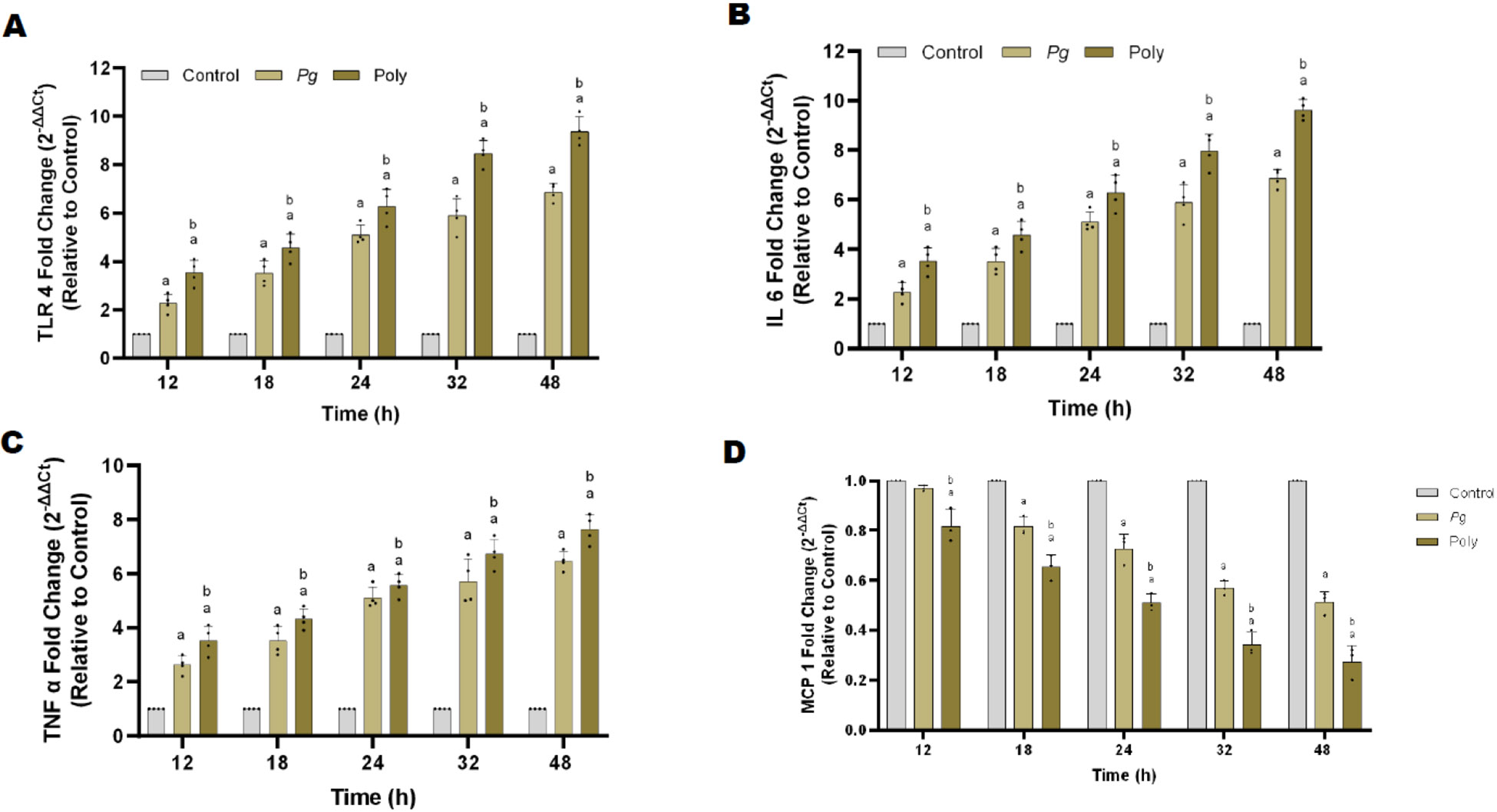
Mono and polybacterial induces inflammatory cytokines in pHAEC’s. **(A)** TLR-4, **(B)** IL-6, **(C)** TNF α and **(D)** MCP-1 at each time point, respectively (0 to 48 h). Data were normalized with housekeeping gene (*β*-actin). All data are represented as 2^−ΔΔCt^ relative to vehicle. The results are from three independent experiments are represented in the form of means ± SD, three independent experiments and, in each experiment, we had duplicates values for all the results). ^*a*^ *p*< 0.05 compared with control (uninfected) cells. ^*b*^ *p*< 0.05 compared with monobacterial infected cells. *Pg*—*P. gingivalis*; Poly – polybacterial; TLR-4 – Toll like receptor 4; IL-6 – interleukin 6; TNF α – tumor necrosis factor alpha; MCP-1 - Monocyte Chemoattractant Protein-1.

The secretion of cytokines by endothelial cells play a crucial role in the onset and advancement of atherosclerosis. Periodontal pathogens exert their pathogenicity through their virulence factors which binds to TLR-4 and activates downstream signaling pathway leading to the secretion of pro-inflammatory cytokines. Here we assessed the gene expression of TLR-4 and downstream signaling cytokines such as Tumor necrosis factor- alpha (TNFα), Interleukin 6 (IL-6), and monocyte chemoattractant protein-1 (MCP-1). Polybacterial infection showed significantly (p<0.05) higher expression (10-fold) of these markers when compared to that of control or *Pg* alone (Fig. 4). In contrast, compared to mono bacterial infection, poly bacterial infection showed a greater reduction in monocyte chemoattractant protein-1 (MCP-1) levels in a time dependent manner (Fig 4D).

### Polybacterial infection decreased antioxidative markers

**Figure 5:**
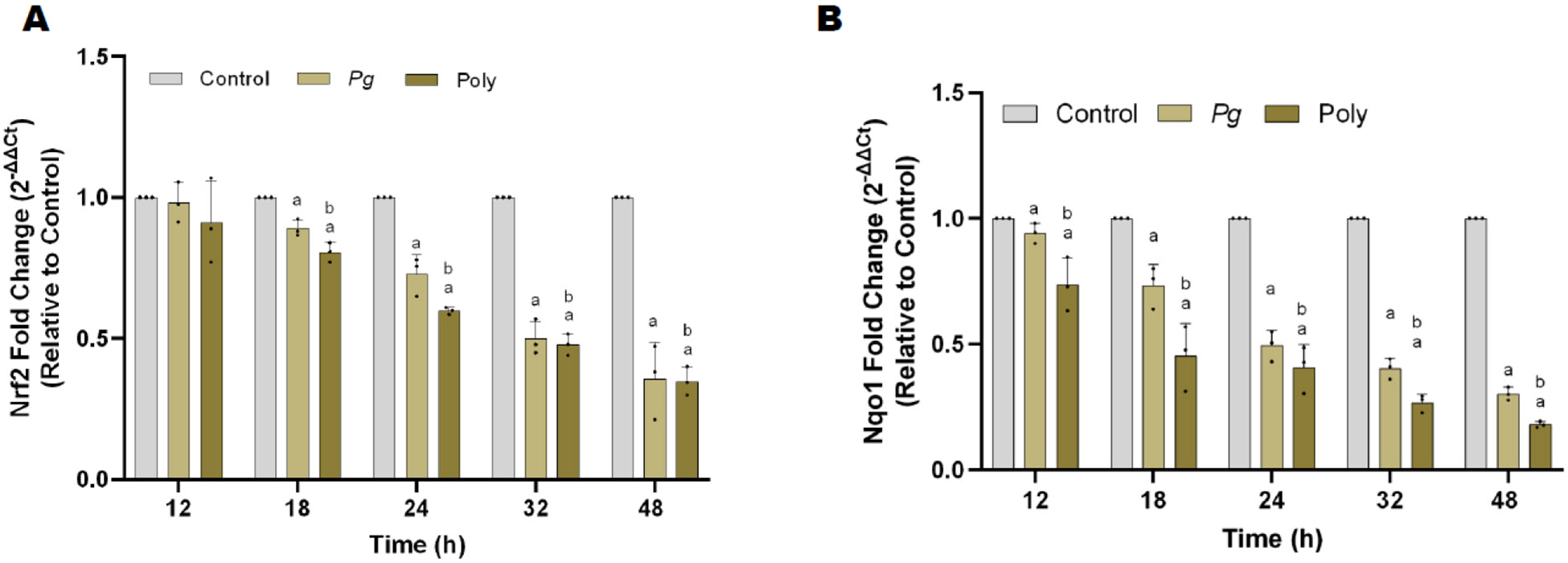
Mono and polybacterial suppresses Nrf2, and NQO1, expression in pHAEC’s. **(A)** Nrf2, and **(B)** NQO1 at each time point (0 to 48 h), respectively. Data were normalized with housekeeping gene (*β*-actin). All data are represented as 2^−ΔΔCt^ relative to vehicle. The results are from three independent experiments are represented in the form of means ± SD, three independent experiments and, in each experiment, we had duplicates values for all the results). ^a^ *p* < 0.05 compared with control (uninfected) cells. ^*b*^ *p*< 0.05 compared with monobacterial infected cells. *Pg*—*P. gingivalis*; Poly – polybacterial; Nrf2 - Nuclear factor erythroid-2 related factor 2; NQO1 - NADPH quinone dehydrogenase.

A direct consequence of the increased production of reactive oxygen species (ROS) is the uncoupling of nitric oxide synthase (NOS) [15]. Nuclear factor erythroid 2 (Nrf2) is a transcription factor that regulates the expression of genes involved in antioxidant defense, detoxification and repair of oxidative damage [16]. NADPH: quinone oxidoreductase 1 (Nqo-1) functions to detoxify and defend cells from offending agents [17]. As shown in figure 5, *Pg* and polybacterial infection decreases both Nrf2 (A) and Nqo-1 mRNA expression (B) in a time-dependent manner.

## Discussion

The vascular endothelium is a single cell layer that lines the entire circulatory system and is viewed as the first line of defense against cardiovascular disease. It has been reported that *Pg* is highly invasive in several cell types, including endothelial cells [18]. The result from our current study clearly shows that monobacterial infection with *Pg* have a deleterious effect on endothelial cell survival only at a later stage than the polybacterial infection (red complex) which had a higher cytotoxicity at earlier time point. This relative pathogenicity between mono-infection and polybacterial culture were influenced by factors like microbial interactions and microbe-host interactions. In an another *in vitro* study, the authors have showed that the invasive abilities of *Pg* enhanced when incubated with *Fn* [19]. Other studies have reported the same differences in oral cavity when mixed or poly- species infections compared to mono- species infection [20-22]. A higher alveolar bone resorption and apical junctional epithelial migration was observed in a rat oral infection model when mixed with *Pg* and *Td* compared to that of mono- infection respectively [21]. In an *in vivo* study, authors have shown that oral infection combined with *Tf* and *Fn* enhanced alveolar bone resorption, osteoclast activation, and gingival tissue leukocyte infiltration than *Tf* or *Fn* alone [22]. In another study polybacterial infection showed transcriptional profiling differences in gene expression in calvarial bone and soft tissue compared to mono- infection with *Pg*, *Td*, and *Tf* [23]. These studies suggest that the host immune and physiologic synergistic interaction exhibit a synergistic interaction when faced with polybacterial infections, which is distinct from the responses observed during individual infections. Collectively, our results suggest that polybacterial infection facilitates invasion of host cells more efficiently than monobacterial infection in pHAEC’s *in vitro*.

Periodontitis-induced endothelial dysfunction is associated with a significant reduction in nitric oxide (NO) bioavailability [4]. NO synthesized by eNOS in the endothelium has been the major source of NO for regulating vaso activity. iNOS is an inducible enzyme that is controlled at the level of gene transcription and is expressed in the immune system in response to inflammatory or proinflammatory mediators. Rosier et al., have reported that when nitrate is added to periodontal plaque, it resulted in nitrite production (an indication of NO production) which in turn reduced periodontitis-associated species, and subgingival microbial dysbiosis index [24]. Our studies in mice infected either with *Pg* or *Td* significantly reduced circulatory NO levels compared to age-matched controls [25-27]. In the same study the authors demonstrated an increased atherosclerotic plaque lesion in mice orally infected with either *Pg* or *Td* [27]. In the current study, polybacterial infection differentially regulates iNOS and eNOS expression. Collectively, our studies suggest that infection with periodontal pathogens contribute to nitrative stress and endothelial dysfunction. Additional studies are warranted to investigate whether polybacterial periodontal infection alters endothelial dependent vascular function *in vivo*. Reduced Tetrahydrobiopterin (BH_4)_ levels in vascular tissue has been reported in coronary artery disease patients causing impaired endothelial function [28]. BH_4_ can be synthesized through de novo and salvage pathways [28]. The de novo BH_4_ synthesis pathway catalyzes guanosine triphosphate (GTP) to the fully reduced biopterin with the rate-liming enzyme GTP cyclohydrolase-1 (GCH-1) [28]. The salvage pathway metabolizes sepiapterin through several reactions, involving sepiapterin reductase (SR) and dihydrofolate reductase (DHFR) [28]. In the absence of BH_4_, eNOS cannot convert L-arginine to nitric oxide, thus uncouples. This uncoupling will result in the production of superoxides or other ROS [28]. ROS oxidizes BH_4_ to dihydropterin (BH_2_), yielding eNOS uncoupling that promotes oxidative stress [28]. An increase in BH_2_ levels result in the decrease of eNOS function, NO synthesis and bioavailability, and oxidative stress regulation [29].

At the initial stages of periodontitis, periodontal pathogens release toxic factors and metabolites [30]. Certain metabolites particularly those acting as damage-associated molecular patterns (DAMPs), recognized by various surface pattern recognition receptors (PRRs) such as NOD-like receptors (NLRs), RI G-I-like receptors (RLRs), C-type lectin receptors (CLRs), and Toll like receptors (TLRs) [31]. TLRs are membrane-bound receptors and are responsible for detecting microbial pathogens and generating innate immune responses [32]. TLR signaling pathways are activated through either MyD88-dependent or MyD88-independent pathway leading to, (1) activating NF-κB and MAPK that enhances the production of pro-inflammatory cytokines, and (2) induction of IFN-β & IFN-inducible genes and maturation of dendritic cells [31]. *Fn* and *Pg* have been shown to exert differential effects at the molecular level on oral epithelial cells and their differences in activating NK-κB nuclear translocation [33]. Secretion of IL-1β, IL-6, IL-8, and RANTES was observed to be increased and differed with the bacterial strain and MOI [34]. TNFα and IL-6 in particular, are associated with diminishing nitric oxide production and endothelial dysfunction, both known precursor factors for atherosclerosis and CVDs [35]. Though we observed in our both experimental groups a significant increase in proinflammatory cytokines, polybacterial infection showed an exponential expression than the *Pg* alone.

Oxidative stress is one of the harmful factor initiating atherosclerosis [36]. Excess production of reactive oxidative species (ROS) causes oxidative stress, leading to cell damage [36]. Increasing evidence indicated that periodontitis induces excessive ROS generation in periodontium [37]. Thus higher ROS generated is dispersed systemically gradually destroying multiple organs [38]. Nuclear factor erythroid-2 related factor 2 (Nrf2), basic leucine zipper transcriptional factor, activates cellular defense mechanism and plays a major role in antioxidant and anti-inflammatory pathways [39]. It has been reported that Nrf2 inhibit the pro-inflammatory effects of TNFα, IL-6, and iNOS expression [40]. We found a significant reduction in Nrf2 expression at each incubation period for both mono- and polybacterial infected cells. This finding is consistent with the periodic increase of TLR-4, IL-6, and TNF α expression in both monobacterial and polybacterial-infected cells. The exponential decline seen in only polybacterial infected pHAECs suggest that Nrf2 is a significant modifier of cytotoxicity and the development of inflammatory diseases [40]. NADPH quinone dehydrogenase (Nqo1), a phase II stress response induced by Nrf2, suppresses ROS production, preventing oxidative stress. Our data aligns with previous studies showing that Nrf2 and Nqo-1 expression are significantly reduced leading to an increased rate of oxidative stress related apoptosis. This further emphasizes the importance of the Nrf2/Nqo-1 pathway in periodontal disease and PD induced cardiovascular complications

## Conclusions

Our data suggests the compared to mono, polybacterial infections significantly alters nitric oxide synthesis, inflammatory and oxidative biomarkers in pHAECs *in vitro*. Although periodontal pathogens act independently of each other, the synchronized effects are more deleterious and detrimental to systemic as well as oral health. Thus, endothelial dysfunction promotes the incidence of systemic inflammatory and autoimmune diseases and disorders. Future studies are warranted to investigate NO mediated vascular function *in-vivo* using murine models infected with mono vs polybacterial pathogens. Furthermore, future research must focus on investigating the unique cytokine signature produced by individual periodontal pathogens as well as their combined effects. Identifying these biomarkers could pave the way for developing new and advanced therapeutic targets to address vascular diseases associated with periodontal disease.

## Figures and Tables

**Table 1:** Primers used for quantitative real-time PCR

Gene	Forward	Reverse
IL-6	5′-TCAATGAGGAGACTTGCCTG-3′	5′- GATGAGTTGTCATGTCCTGC-3′
MCP-1	5′-GAAAGTCTCTGCCGCCCTT-3′	5′- TTGATTGCATCTGGCTGAGCG-3′
TLR-4	5′-GATCTGTCTCATAATGGCTTG-3′	5′- GACAGATTCCGAATGCTTGTG-3′
TNF α	5′- CACGCTCTTCTGTCTACTGA-3′	5′-ATCTGAGTGTGAGGGTCTGG-3′
β-actin	5′-ATCCTCACCCTGAAGTACCC-3′	5′-TAGAAGGTGTGGTGCCAGAT-3′
e-NOS	5′-TACGCACCCAGAGCTTTTCT-3′	5′-CTTGGTCAACCGAACGAAGT-3′
iNOS	5′-CTTCAACCCCAAGGTTGTCTGCAT-3′	5′-ATGTCATGAGCAAAGGCGCAGAAC-3′
GCH-1	5′- GAGCATCACCTTGTTCCATTTG -3′	5′- GCCAAGTTTACTGAGACCAAGGA -3′
Nrf2	5′-TCTCCTCGCTGGAAAAAGAA-3′	5’-TAAAGCACAGCCAGCACATT-3’
Nqo1	5'- GCT GGAACAGGAAGAAGAAG -3'	5'- GCA GGAGGAAGAAGAAGGA -3’
